# Reliability and Time Course of Postexercise Hypotension during Exercise Training among Adults with Hypertension

**DOI:** 10.3390/jcdd11020042

**Published:** 2024-01-29

**Authors:** Peter A. Kiernan, Christina A. Day, Rachel S. Berkowsky, Amanda L. Zaleski, Simiao Gao, Beth A. Taylor, Lucas P. Santos, Gregory Panza, Melody Kramarz, Kyle McCormick, Paul D. Thompson, Antonio B. Fernandez, Ming-Hui Chen, Linda S. Pescatello

**Affiliations:** 1 Department of Kinesiology, University of Connecticut, Storrs, CT 06269, USA; christina.day@uconn.edu (C.A.D.); rachel.berkowsky@uconn.edu (R.S.B.); simiao.gao@uconn.edu (S.G.); melody.kramarz@uconn.edu (M.K.); kyle.mccormick@uconn.edu (K.M.); ming-hui.chen@uconn.edu (M.-H.C.); linda.pescatello@uconn.edu (L.S.P.); 2 Hartford Hospital, Hartford, CT 06102, USA; zaleskia@aetna.com (A.L.Z.); beth.taylor@cigna.com (B.A.T.); gregory.panza@hhchealth.org (G.P.); pthompson@hhchealth.org (P.D.T.); antonio.fernandez@hhchealth.org (A.B.F.); 3 Hospital de Clínicas de Porto Alegre, Porto Alegre 90035-903, Brazil; lucaspsantos87@gmail.com

**Keywords:** blood pressure, cardiovascular disease, physical activity, antihypertensive therapy

## Abstract

Postexercise hypotension (PEH), or the immediate decrease in blood pressure (BP) lasting for 24 h following an exercise bout, is well-established; however, the influence of exercise training on PEH dynamics is unknown. This study investigated the reliability and time course of change of PEH during exercise training among adults with hypertension. PEH responders (*n* = 10) underwent 12 weeks of aerobic exercise training, 40 min/session at moderate-to-vigorous intensity for 3 d/weeks. Self-measured BP was used to calculate PEH before and for 10 min after each session. The intraclass correlation coefficient (ICC) and Akaike Information Criterion (AIC) determined PEH reliability and goodness-of-fit for each week, respectively. Participants were obese (30.6 ± 4.3 kg∙m^−2^), middle-aged (57.2 ± 10.5 years), and mostly men (60%) with stage I hypertension (136.5 ± 12.1/83.4 ± 6.7 mmHg). Exercise training adherence was 90.6 ± 11.8% with 32.6 ± 4.2 sessions completed. PEH occurred in 89.7 ± 8.3% of these sessions with BP reductions of 9.3 ± 13.1/3.2 ± 6.8 mmHg. PEH reliability was moderate (ICC ~0.6). AIC analysis revealed a stabilization of maximal systolic and diastolic BP reductions at 3 weeks and 10 weeks, respectively. PEH persisted throughout exercise training at clinically meaningful levels, suggesting that the antihypertensive effects of exercise training may be largely due to PEH. Further studies in larger samples and under ambulatory conditions are needed to confirm these novel findings.

## 1. Introduction

Hypertension affects 46.7% of adults in the United States and is a major risk factor for cardiovascular disease, the number one cause of death in the United States and world [1]. The direct and indirect medical costs of cardiovascular disease in the United States totaled $497.3 billion from 2018 to 2019 and are projected to increase to $1.1 trillion by 2035 [1,2]. Aerobic and resistance exercise training alone or combined reduce resting blood pressure (BP) by 5–8 mmHg among adults with hypertension [3,4]. BP reductions of this magnitude reduce the risk of cardiovascular disease mortality by 16%, substantiating the vital role of exercise as an antihypertensive lifestyle therapy [5].

Exercise also elicits immediate BP reductions of 5–8 mmHg following a single bout of exercise that persist for up to 24 h, referred to as *postexercise hypotension* (PEH) [6,7]. PEH is clinically important because those with the highest resting BP, i.e., adults with hypertension, experience the greatest BP reductions [8]. PEH is directly correlated with the magnitude of the BP reductions that occur with more long-term exercise training among adults with hypertension, suggesting that it may be used as a screening tool to predict who will be responsive to more long-term exercise training as antihypertensive lifestyle therapy [9,10]. Furthermore, some have postulated that PEH may account for some, if not all, of the BP reductions resulting from exercise training [11,12]. Nonetheless, questions remain as to how exercise training impacts the magnitude of PEH and the consistency by which PEH manifests itself during exercise training.

The studies investigating the influence of exercise training on PEH have yielded mixed results. Moraes et al. concluded that PEH was attenuated by ~17 mmHg for systolic BP (SBP) and ~9 mmHg for diastolic BP (DBP) following 12 weeks of dynamic resistance training among men with hypertension [13]. Similarly, Mota et al. found that PEH first appeared by month two of a 16 weeks dynamic resistance training program but was attenuated by ~6 mmHg for SBP at month three and by ~4 mmHg for DBP at month four of the resistance training program among older women with hypertension [14]. Iellamo et al. found that PEH did not occur as assessed by 24 h ambulatory BP (ABP) following a bout of continuous aerobic exercise after 12 weeks of aerobic exercise training but did manifest itself following a high-intensity interval exercise session performed after the same 12 weeks of aerobic exercise training among older adults with hypertension [15]. Imazu et al. reported that PEH was greater, as assessed by 12 h ambulatory BP, by ~5 mmHg following several months of aerobic exercise alone or combined with resistance exercise training among adults with hypertension compared to sedentary controls [16]. Due to differences in these study designs [13,14,15,16], the BP and medication status of the samples, and BP assessment methods, no definitive conclusions can be made regarding whether PEH is unaffected, attenuated, or augmented by exercise training.

In the few studies that have quantified the reliability of PEH between exercise sessions, PEH appears to be reliably produced. In a pair of studies each employing an acute test and retest protocol comprised of a control and moderate-intensity aerobic exercise session repeated twice, Fecchio et al. reported that PEH had moderate to excellent reliability for SBP and poor reliability for DBP [17], as well as good within-individual consistency for mean arterial BP [18], among 25 and 30 adults with normal BP, prehypertension (i.e., elevated BP), and hypertension. Fonseca et al. found that PEH occurred with moderate to excellent reliability for SBP and good to excellent reliability for DBP across a single control visit and two moderate-intensity mixed-circuit exercise sessions performed in a random order among a sample of adults recovering from stroke [19]. Surprisingly, the reliability of PEH throughout more long-term aerobic exercise training, to the best of our knowledge, has not been investigated.

The purpose of this study was to determine the reliability of PEH throughout a 12 weeks moderate-to-vigorous intensity aerobic exercise training program among adults with hypertension. We hypothesized that PEH would demonstrate moderate reliability before, during, and after aerobic exercise training. As a secondary aim, we sought to characterize the time course of the pattern of the change in PEH magnitude for each week of the aerobic exercise training program.

## 2. Materials and Methods

The present sub-study is part of a larger study, *Blood Pressure Utilizing Self-Monitoring after Exercise* or PULSE (ClinicalTrials.gov Identifier NCT03780309) [20]. For this reason, we performed a post hoc power analysis later described in the statistical analysis section. Participants indicated their willingness to participate by signing an informed consent form approved by the Institutional Review Boards of the University of Connecticut and Hartford Hospital.

### 2.1. Participants

Participants were ≥18 years of age with hypertension recruited through flyers, newsletters, and social media postings from October 2016 to May 2018. To be included, participants were (1) free of diagnosed chronic diseases (e.g., cardiovascular disease, metabolic syndrome, and depression); (2) consumed less than two alcoholic drinks a day; (3) nonsmokers for 6 months prior to study entry; and (4) physically inactive, defined as performing formal exercise less than 2 d/week [21]. Participants were excluded if they (1) were planning to become pregnant and/or lactating; (2) presented with orthopedic conditions that interfered with their ability to exercise; (3) had a history of cancer-related lymphedema; (4) intended to gain or lose weight; and (5) had a resting SBP ≥ 160 mmHg and/or DBP ≥ 100 mmHg [22]. Participants who were taking medication or supplements that affected BP were asked to discontinue use prior to entry and for the duration of the study upon receiving the prescribing provider’s consent.

### 2.2. Study Overview

**Figure 1:**
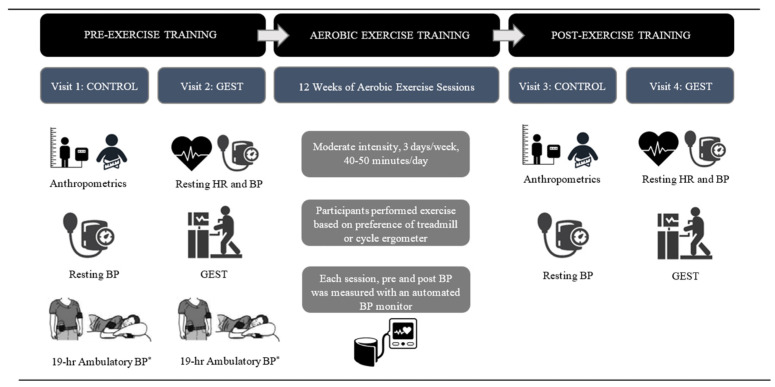
Study design overview. ABP = ambulatory blood pressure; BP = blood pressure; GEST = graded exercise stress test; HR = heart rate. * To identify PEH responders for study inclusion.

The study overview is shown in Figure 1. This sub-study investigated the reliability of PEH over 12 weeks of supervised exercise training among a subsample of six male and four female participants from the larger study. PEH was defined as a decrease in SBP and/or DBP of any magnitude post-exercise versus pre-exercise [23]. All study visits occurred at Hartford Hospital by the same trained investigator, at the same time of day, and were separated by at least 48 h.

### 2.3. Visit 1 and 2: Control Visit and Graded Exercise Stress Test Visit

Participants reviewed and signed the informed consent form at their first study visit. All but one participant (*n* = 9 out of 10) completed a control session (CONTROL) on Visit 1 before the maximal cardiopulmonary graded exercise stress test (GEST) on Visit 2. Participants fasted overnight and abstained from consuming alcohol and caffeine before Visits 1 and 2. At the conclusion of Visits 1 and 2, participants were fitted with an ABP monitor on the non-dominant arm. Visits 1 and 2 were completed in the morning at approximately the same time of day. The ABP data from Visits 1 and 2 were used to determine whether participants were confirmed as PEH responders, defined as having an average 24 h ambulatory SBP and/or DBP that is ≥2 mmHg lower after GEST than after CONTROL [20].

#### 2.3.1. Control

Participants completed CONTROL to account for the circadian variation in BP [23] and the confounding effects of the laboratory on BP [24]. During CONTROL, we measured body weight, height, and waist circumference. Resting BP was measured by oscillation according to American Heart Association (AHA) guidelines [4] using a BPTRU monitor (BPTRU Medical Devices; Coquitlam, BC, Canada). Participants sat for 15 min and then resting BP was measured three times at five-minute intervals on each arm. These values were averaged and recorded as resting BP. Resting BP measurements were performed by the same study investigator to control for intertester variability.

#### 2.3.2. Maximal Graded Exercise Stress Test

During the GEST, participants completed a maximal cardiopulmonary GEST using the Balke protocol [25]. At the beginning of this visit, the same investigator measured resting BP using the same procedures as during CONTROL. Resting heart rate (HR) was measured in the supine position following a 5 min rest period using the GE Case Exercise Testing ECG System (GE Healthcare, Wauwaposa, WI, USA). Immediately prior to the GEST, the study physician or their designee performed a brief physical examination. During the GEST, the physician monitored the participants’ electrocardiogram and other physical signs. After the GEST was completed, the study physician reviewed the electrocardiogram. We determined peak oxygen uptake (VO_2_peak) by breath-by-breath analysis of expired gases (i.e., oxygen and carbon dioxide) (ParvoMedicsTrueOne^®^ 2400 Metabolic Measurement System, ParvoMedics Inc., Sandy, UT, USA). We measured HR continuously with a 12-lead electrocardiogram system and BP by auscultation every 3 min during the GEST. The maximal HR (HR_max_) recorded during the GEST was used along with resting HR to establish and monitor exercise intensity during the supervised exercise training program.

### 2.4. Exercise Training and Self-Monitoring of Exercise

After Visits 1 and 2, participants completed a 12 week supervised aerobic exercise training program, 3 d/week at a moderate-to-vigorous intensity. In addition, participants were encouraged to perform unsupervised exercise sessions at home for at least 30 min/d, 1–2 d/week. Participants were asked to continue their usual dietary and lifestyle habits for the duration of the exercise training program. Prior to exercise training, participants received a home BP monitor (Omron MEM-705CPN; Omron Healthcare, Bannockburn, IL, USA) and were trained to use it by investigators [4]. During both supervised and unsupervised exercise sessions, participants self-measured resting BP with a home BP monitor on their non-dominant arm three times at 1 min intervals after 5–10 min of seated rest pre- and post-exercise. Participants were educated on how to document their pre- and post-exercise resting BP measurements with the AHA “Check. Change. Control. Tracker” BP monitoring tool [26]. During exercise training, participants self-monitored exercise using a diary recording method [27], by recording the frequency, intensity, time, and type of each exercise training session. Average HR during each exercise session, the rating of perceived exertion on the Borg 6–20 scale [28], and training impulse [29] were recorded for all supervised and unsupervised exercise sessions.

### 2.5. Visits 3 and 4: Post-exercise Training Control and GEST

Following the completion of exercise training, nearly all but two participants (*n* = 8 out of 10) completed the post-exercise training CONTROL (Visit 3) before the GEST (Visit 4) visit. Visits 3 and 4 were conducted within 24–72 h of the last training session to account for the confounding BP effects of acute exercise and detraining [10]. Visits 3 and 4 were performed according to the same protocols and at approximately the same time of day as Visits 1 and 2.

### 2.6. Measurement Protocols

#### 2.6.1. Body Composition

During both CONTROL visits (Visits 1 and 3), height and weight were measured with a calibrated balance beam scale to calculate body mass index (BMI) (kg/m^2^). Waist circumference (cm) was measured at the iliac crest using a non-distensible Gulick tape measure [21]. During the exercise training program, participants were weighed monthly to ensure weight maintenance.

#### 2.6.2. Ambulatory Blood Pressure

At the conclusion of Visits 1 and 2, we fitted the participants with an Oscar2 automatic noninvasive ABP monitor (Suntech Medical Instruments Inc., Raleigh, NC, USA) following standard protocols via a calibration check with a t-tubule. The same ABP monitor was worn by the same participants for both Visits 1 and 2. The ABP monitor was programmed to record BP and HR at regular intervals three times per waking hour and two times per sleeping hour. Participants were instructed to proceed with normal activities, not to exercise, and to keep their arm still and extended at their side while measurements were being taken by the ABP monitor. Participants were given a journal to carry with them in which they recorded any activity performed during each measurement, any abnormal physical or emotional events, and sleep and wake times. Participants detached the monitor and returned it to the study investigators the following morning.

Computerized ABP reports were considered acceptable if at least 80% of the BP readings were obtained. ABP measurements of SBP > 220 and/or DBP > 130 mmHg were omitted according to the manufacturer’s exclusion criteria. The ABP data were imported using the Accuwin Pro v3 software for statistical analyses.

#### 2.6.3. Heart Rate and the Intensity of Exercise

Resting HR and HR_max_ from the pre-exercise training GEST (Visit 2) were used to calculate the HR reserve for each participant as [(HR_max_ − resting HR) × 40 to 60%] + resting HR [21] to set the target exercise intensity. Prior to the exercise training, participants were trained to use the polar FT7 HR monitor (Polar Electro Oy, Kempele, Finland) to record HR during each supervised and unsupervised exercise session. The HR monitor was worn on the chest with a strap and was equipped with coded HR transmission and graphical target HR zone displays on a wearable wrist-worn watch. Study investigators used the exercise HR data to determine several measures of exercise intensity including % HR_max_, % HR reserve, and the training impulse of each exercise session [21]. The training impulse was calculated as the duration of the session × ΔHR ratio × Y, where ΔHR ratio was (exercise HR − resting HR/HR_max_ − resting HR) and Y was 0.64e^(1.92 × ΔHR ratio)^ for men and 0.86e^(1.67 × ΔHR ratio)^ for women [29].

#### 2.6.4. Self-Monitoring of the Frequency, Intensity, Time, and Type of Exercise

Prior to exercise training, participants were trained to use a validated diary recording method, the Timeline Follow Back for Exercise [27], to record the frequency, intensity, time, type, and rating of perceived exertion on the Borg 6–20 scale [28} of the exercise they performed over the 12 weeks of exercise training for both the supervised and unsupervised sessions. The frequency of exercise was the number of exercise sessions performed during each week of exercise training. The intensity of each exercise session was recorded as the % HR_max_, % HR reserve, and training impulse. In addition, the Borg rating of perceived exertion was recorded every 5 min during each exercise session and averaged for each exercise session. The time of exercise was measured as the duration of each exercise session in minutes. For the type of exercise, participants had the option to perform either walking, jogging, or cycling at each session. The total number of exercise sessions performed using each modality was recorded for each participant as [(number of sessions on treadmill or stationary bike/total number of sessions performed) × 100%]. All training logs were returned weekly to investigators for review.

### 2.7. Statistical Analyses

Baseline descriptive characteristics, the average frequency, intensity, time, and type of exercise achieved during exercise training, and changes in BP from pre- to post-exercise were calculated in Microsoft Excel version 2208 (Microsoft Corporation, Redmond, WA, USA) and reported as mean ± standard deviation for the sample. To determine the occurrence of PEH throughout the 12 weeks of exercise training, we used the difference in post- minus pre-exercise resting BP from the three weekly supervised sessions per participant for each week for SBP and DBP [23]. We also calculated the relative percent change in BP as post- minus pre-exercise BP divided by pre-exercise BP × 100% for each exercise training session for SBP and DBP. A negative percent change in SBP and/or DBP from baseline indicated that PEH occurred. If a participant performed less than three supervised exercise sessions in each week, and if BP data from the unsupervised session(s) performed in that same week were available, we used the unsupervised session(s) BP data to replace the missing supervised exercise session(s) BP data from that week for as many unsupervised sessions available for the supervised sessions not performed. The missing supervised exercise session BP data were replaced for each of four participants as follows: (1) the missing BP data from three supervised sessions with three unsupervised exercise sessions; (2) the missing BP data from two supervised sessions with two unsupervised exercise sessions; and (3) for two participants, the BP data from one supervised session with one unsupervised exercise session. We report exercise adherence as [(average number of sessions completed/the total number of possible supervised sessions) × 100%].

#### 2.7.1. Reliability Statistical Analysis

We determined the reliability of the occurrence of PEH during the 12 weeks of aerobic exercise training via two methods: (1) a continuous PEH outcome variable defined as the lowest SBP or DBP percent relative change ([post-exercise − pre-exercise/pre-exercise] × 100%) for each exercise session; and (2) a binary PEH outcome variable defined as a decrease in BP post- vs. pre-exercise for each exercise session, “Yes” or “No”.

For the continuous PEH outcome variable, we derived the between-participant and within-participant variability from a repeated-measures analysis of variance (RMANOVA) [30] with training week as a factor variable and a repeated-measures analysis of covariance (RMANCOVA) [31] with training weeks as a factor variable adjusted for pre-exercise SBP and DBP as covariates. The intraclass correlation coefficient (ICC) was calculated as a ratio of the between-participant variability ($\sigma_{b}^{2}$) and the sum of the between-participant variability and within-participant variability ($\sigma_{e}^{2}$) for the BP outcomes (ICC =$/\left(\sigma_{b}^{2}+\sigma_{e}^{2}\right)$) (see Supplemental Formulas S1, which demonstrate the determination of the ICC).

For the binary PEH outcome variable, ICC was calculated in a similar way as for the continuous PEH response. However, we extended a method by Dimitrov [32] for the repeated binary PEH responses to compute the between-participant variability and within-participant variability based on the estimated probability of PEH for each week. The between-participant variability and within-participant variability used to calculate the ICC for the binary PEH response were derived from a generalized repeated-measures analysis of variance (GRMANOVA) model with training week as a factor variable and a generalized repeated-measures analysis of covariance (GRMANOVA) model with training week as a factor variable adjusted for pre-exercise BP. The ICC values of <0.5, ≥0.5 and ≤0.75, >0.75 and ≤0.90, and >0.90 are indicative of poor, moderate, good, and excellent reliability, respectively [33].

We used the Akaike Information Criterion (AIC) to compare the RMANOVA and RMANCOVA models as well as the GRMANOVA and GRMANCOVA models (see supplemental formulas for the calculation of the AIC). The AIC values indicated that the RMANCOVA (AIC = 2177.9; SBP: Chi-squared = 49.6, *p* = 0.00; DBP: Chi-squared = 3.7, *p* = 0.05) and GRMANCOVA (AIC = 224.9; SBP: Chi-squared = 7.8, *p* = 0.005; DBP: Chi-squared = 0.06, *p* = 0.8100) were statistically superior, so we only report the ICC values for the models with covariates in our results (see Supplemental Table S1 for a comprehensive comparison of the results for each model). We further used the residuals to check the goodness-of-fit of the RMANCOVA and GRMANCOVA models. A *p*-value of ≤0.05 was considered statistically significant.

#### 2.7.2. The Post Hoc Power Analysis

We performed a post hoc power analysis to test the hypothesis, $H_{0}:\;\mathrm{ICC}\;\leq \;0.5\;\mathrm{vs}.{\;H}_{a}:\;\mathrm{ICC}\;>0.5$, and calculated the power of the test. We simulated 5000 datasets based on the between-participant variability ($\sigma_{b}^{2}=51.63$) and measurement error variability ($\sigma_{e}^{2}=36.86$) while keeping the covariates constant. Binary PEH outcomes were determined according to the simulated continuous BP outcomes. Furthermore, RMANCOVA and GRMANCOVA models were performed on each simulated dataset. The resulting power of the test for the RMANCOVA and GRMANCOVA models was 0.651 and 0.949, respectively.

#### 2.7.3. The Magnitude of PEH during Exercise Training Statistical Analysis

To answer the question of whether the magnitude of PEH changes or remains the same as weeks of exercise training progress, we assumed that the means of PEH changes were constant since the jth week for 1≤j≤12 and used the RMANCOVA model with the modified week variable, that was, week 1, week 2, …, week j to week 12, as a factor variable and pre-exercise SBP and DBP as covariates. To determine the goodness of fit, we used the AIC to compare the 12 models described above and identify the model that fit the data best. The models for SBP and DBP change are defined below:

$$\mathrm{Model}\;m\;(1\leq m\leq 12):\;y_{ijk}=\gamma x_{ijk}+\overset{m-1}{\underset{p=1}{\sum }}\beta_{p}1\left(j=p,m>1\right)+\beta_{m}1\left(m\leq j\leq 12\right)+\lambda_{i}+\varepsilon_{ijk},\;i=1,...,10;\;j=1,...,12;\;k=1,...,3.$$

where $y_{ijk}$ was the SBP or DBP change, $x_{ijk}$ was the corresponding baseline measure, and γ denoted the corresponding coefficient of $x_{ijk}$ for the ith participant in the jth week and kth session. Moreover, $\beta_{p}$ denoted the fixed effect for the pth week and $\beta_{m}$ denoted the constant effect for weeks since the mth week, which means that we assumed the means of week m through week 12 were the same. The variable $1\left(j=p,m>1\right)$ took the value 1 under the condition when *j* = *p* and *m* > 1, 0 otherwise and $1\left(m\leq j\leq 12\right)$ was defined similarly. In addition, let $\lambda_{i}$ be the random effect for the ith participant and $\varepsilon_{ijk}$ be the measurement error term. We assumed that

$$\lambda_{i}\;\sim \;N\left(0,\tau^{2}\right),\;\varepsilon_{ijk}\;\sim \;N\left(0,\sigma^{2}\right).$$

We used model 3 (*m* = 3) to illustrate the model. When *j* = 1, $E(y_{ijk})=\gamma x_{ijk}+\beta_{1}.$ When j = 2, $E(y_{ijk})=\gamma x_{ijk}+\beta_{2}.$ When 3≤j≤12, $E(y_{ijk})=\gamma x_{ijk}+\beta_{3}$.

Statistical analyses and other calculations were performed in R (version 4.0.2, R Core Team (2020)), Microsoft Excel version 2208 (Microsoft Corporation, Redmond, WA, USA), and SPSS Statistics version 25.0 (IBM Corporation, Armonk, NY, USA). The models were fitted with the package lme4 [34], and the package ggplot2 [35] was used to plot figures. ANOVA tables were generated with the package car [36], and data cleaning and manipulation were conducted with the package dplyr [37]. The R-codes for fitting the models and computing AIC and ICC (see Supplemental Codes S1), as well as the residual plots (see Supplemental Figures S1 and S2), are in the Supplementary Materials.

## 3. Results

### 3.1. Participant Characteristics Pre-Exercise Training

Participant characteristics are shown in Table 1. Participants (*n* = 10) were middle-aged Caucasian men (60%) and women with stage I obesity [21] and stage I hypertension [4]. Based on the age- and sex-specific normative values, men had “good” cardiorespiratory fitness and women had “fair” cardiorespiratory fitness [21]. Participant disease risk for type 2 diabetes mellitus, hypertension, and cardiovascular disease based on their BMI and waist circumference values was “high” for men and “very high” for women. Of the participants who were taking antihypertensive medications, three were on angiotensin II receptor blockers and one was on an angiotensin-converting enzyme inhibitor.

### 3.2. Exercise Training Program

The 12 week aerobic exercise training program consisted of three sessions/week for 36 min/session at moderate-to-vigorous intensity (Table 2) [21]. Out of a possible 36 exercise sessions, the average adherence was 90.8 ± 11.9% of the sessions. Most exercise sessions were performed on a treadmill with the remainder performed on a stationary bike. There were no exercise-related injuries reported during the study period.

### 3.3. Participant Characteristics Post- versus Pre-Exercise Training

Resting SBP decreased 8.8 ± 9.7 mmHg (*p* = 0.019) and DBP decreased 5.5 ± 7.5 mmHg (*p* = 0.044) post- versus pre-exercise training, which resulted in the sample BP classification improving from stage I hypertension to elevated BP [4]. VO_2_peak increased 4.2 ± 4.3 mL·kg^−1^·min^−1^ (*p* = 0.013), resulting in the cardiorespiratory fitness classification improving from “fair” to “excellent” among the women with no change in the men over the course of exercise training [21]. BMI, waist circumference, and resting HR were not different post- versus pre-exercise training (*p* > 0.05).

### 3.4. The Magnitude, Reliability, and Time Course of PEH during Exercise Training

**Figure 2:**
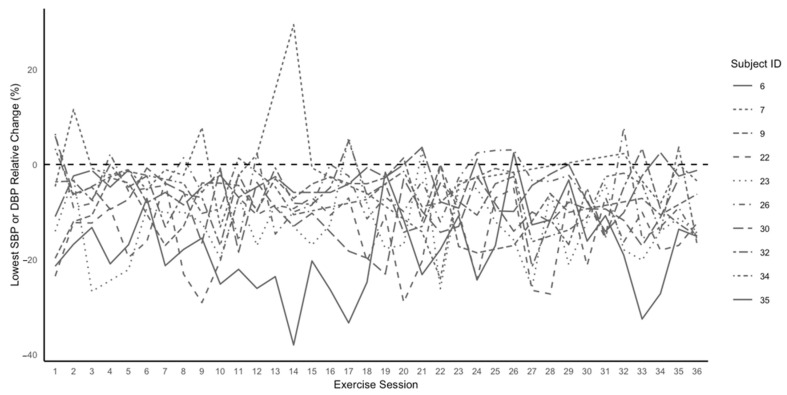
The manifestation of PEH as the lowest % change for systolic or diastolic blood pressure after versus before each exercise session for each participant over 12 weeks of exercise training. SBP, systolic blood pressure; DBP, diastolic blood pressure.

**Figure 3:**
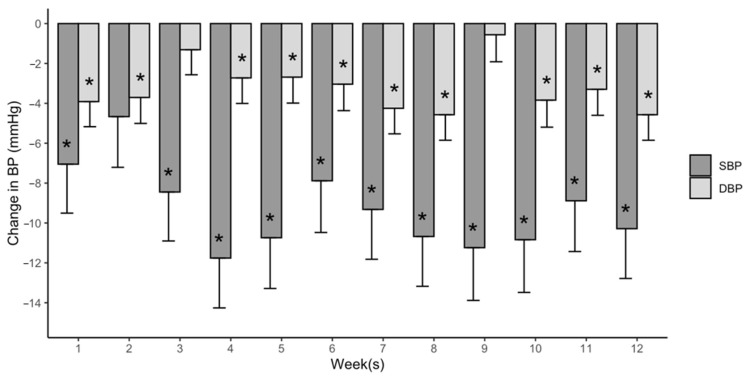
The average magnitude of PEH for each week of exercise training (mean + SE). BP, blood pressure; SBP, systolic blood pressure; DBP, diastolic blood pressure. * *p* < 0.05

Figure 2 displays the manifestation of PEH by the lowest % change in SBP or DBP for each participant after versus before each exercise session over 12 weeks. PEH occurred in 89.4 ± 8.7% of the 32.7 ± 4.3 completed exercise sessions, averaging a decrease of 9.3 ± 13.1 mmHg for SBP and 3.2 ± 6.8 mmHg for DBP over 12 weeks. The ICC values for the manifestation of PEH in the RMANCOVA (0.58) and GRMANCOVA (0.64) models for each exercise session over 12 weeks indicated that PEH occurred with moderate reliability [35]. The AICs from the RMANCOVA assessed the time course of the manifestation of PEH and indicated that the decrease in SBP maximized by 3 weeks, as the SBP decreases were not different after 3 weeks (*P*s > 0.05), whereas DBP maximized by 10 weeks, as the DBP decreases were not different after 10 weeks (Figure 3).

## 4. Discussion

The primary purpose of this study was to characterize the reliability of the occurrence of PEH during a 12 week aerobic exercise training program among adults with hypertension. A secondary purpose was to evaluate the time course of change in the magnitude of PEH during the exercise training program. Our new findings show that PEH occurred in nearly 90% of the exercise sessions performed for 3 d/week for 12 weeks with average BP reductions of 9 mmHg for SBP and 3 mmHg for DBP while demonstrating moderate reliability. In addition, the magnitude of PEH maximized by 3 weeks of exercise training for SBP and did not differ thereafter, while the magnitude of PEH maximized by 10 weeks of exercise training for DBP and did not differ thereafter. Thus, PEH was not attenuated by exercise training as some have proposed [13] but rather is an important antihypertensive lifestyle therapy in and of itself, contributing to some if not all the antihypertensive effects of more long-term exercise training as we and others have posited [11,12]. Indeed, the reductions in BP we observed of 3 to 9 mmHg because of PEH correspond to reductions in the risk of cardiovascular disease mortality by 16%, 18% for coronary heart disease, and 36% for stroke, and they match the magnitude of BP reductions reported for antihypertensive medications [5].

To our knowledge, we are the first to investigate the reliability of PEH during long-term aerobic exercise training among adults with hypertension. Consistent with our findings, several research teams have investigated the reliability of PEH across a small number of exercise sessions and found PEH to be reliable. Fonseca et al. examined the reliability of PEH across a control and two mixed-circuit exercise sessions among adults with hypertension recovering from stroke [19]. They found that the reliability of PEH was moderate to excellent for SBP and DBP. Likewise, Fecchio et al. found that PEH had moderate to excellent reliability for SBP during a test and retest protocol comprised of a control and aerobic exercise session repeated twice among adults with normal BP, prehypertension, and hypertension; however, they found poor reliability for DBP [17]. In a more recent study, Fecchio et al. used the same test and retest protocol among adults with normal BP, prehypertension, and hypertension and found that the consistency of PEH was good for mean arterial BP but marginal for SBP and DBP [18]. We expand upon these findings of the reliability of PEH over a few exercise sessions to 12 weeks of exercise training, reinforcing the clinical relevance of PEH as an antihypertensive lifestyle therapy.

This study was not designed to investigate the mechanisms underlying PEH, and therefore, it is unclear what underlies the consistency by which PEH occurred during exercise training in our study. PEH occurs through a decrease in cardiac output and/or peripheral vascular resistance, which has been reported to be mediated by central (e.g., baroreflex adjustments, decreases in sympathetic input to the heart and vasculature, decreased stroke volume) and/or peripheral mechanisms (e.g., decreased vasoconstrictor molecules, increased release of local vasodilator molecules) [12,38,39]. Fecchio et al. are one of the few that investigated the consistency of PEH and associated mechanisms during a control and aerobic exercise session repeated twice in an acute test and retest design among adults with normal BP, prehypertension, and hypertension [18]. They found that following both exercise sessions, compared to the control, PEH occurred for mean arterial BP in 70% of participants, but the associated hemodynamic responses including measures of heart rate variability, baroreflex sensitivity, systemic vascular resistance, and cardiac output were variable between the exercise sessions. They concluded that different mechanisms may lead to PEH on two different exercise days within the same individual. The inclusion of participants with mixed BP status and the small number of sessions limits the applicability of their observations to our study and any conclusions we can make regarding mechanisms behind the reliability of PEH throughout exercise training.

Several studies have investigated whether PEH is augmented, unaffected, or attenuated by aerobic exercise training. Mota et al. and Moraes et al. found that the magnitude of PEH diminished during a 16 week [14] and 12 week [13] dynamic resistance training program performed by adult men and older women with hypertension. Iellamo et al. investigated PEH following a single bout of dynamic resistance, high-intensity interval, and continuous aerobic exercise before and after a 12 week aerobic training program. They found PEH assessed by 24 h ABP monitoring was attenuated after a session of dynamic resistance exercise and a session of continuous aerobic exercise but not after a session of high-intensity interval exercise versus before 12 weeks of aerobic exercise training [15]. Imazu et al. found that PEH assessed for 12 h under ambulatory conditions was augmented by ~5 mmHg after 4 months of an aerobic or concurrent exercise training program among adults with controlled hypertension [16]. Differences in the study designs including exercise modalities, BP assessment methods, and the BP status of study participants limit any conclusions that can be made on the influence of exercise training on the magnitude of PEH from these studies.

Our findings on the time course of changes in PEH magnitude suggest that PEH is not attenuated during aerobic exercise training among adults with hypertension. The magnitude of PEH for SBP stabilized at 3 weeks of exercise training and did not statistically differ thereafter. The average reduction in SBP was ~7 mmHg for the first 3 weeks and ~10 mmHg for the last 9 weeks of exercise training and averaged ~9 mmHg over the 12 week training program. The magnitude of PEH for DBP stabilized at 10 weeks and did not differ thereafter. The average reduction in DBP was ~3 mmHg for the first 9 weeks and ~4 mmHg for the last 3 weeks of training and averaged ~3 mmHg over the 12 weeks of exercise training. Our findings suggest aerobic exercise training may augment PEH initially, and the magnitude of PEH then stabilizes at clinically meaningful levels as aerobic exercise training progresses among adults with hypertension.

Our study has several limitations. It consisted of a small sample of mostly Caucasian men with hypertension. It was a sub-study and post hoc analysis of a larger study [20] that enrolled PEH responders defined as exhibiting a decrease in 24 h ambulatory SBP and/or DBP of 2 mmHg or more following a GEST versus a non-exercise control session. Thus, our findings are not generalizable to PEH non-responders. Nonetheless, PEH has been reported to not occur in ~25% of people for reasons that are not clear, which indicates that PEH non-responders represent a small proportion of people with hypertension [40]. During the exercise training sessions, BP was measured at 5–10 min pre- and post-exercise in the laboratory. Determining how long PEH was sustained under ambulatory conditions following each aerobic exercise training session was not possible and is a study limitation [23]. The strengths of our study include the rigorous study design [24]; the participants having hypertension, so they were not of mixed BP status; and PEH being assessed throughout 12 weeks of aerobic exercise training.

## 5. Conclusions

Our novel finding is that PEH occurred in 90% of exercise training sessions with moderate reliability. The average magnitude of the PEH reductions was 9 mmHg for SBP and 3 mmHg for DBP. Our findings suggest that PEH is not attenuated by exercise training but occurs at clinically relevant levels throughout exercise training, reinforcing its importance as an antihypertensive lifestyle therapy. Future studies with larger and more diverse samples are needed to confirm our promising findings among PEH responders and non-responders alike.

## Figures and Tables

**Table 1:** Participant characteristics prior to exercise training, *n* = 10 (Mean ± SD).

Age (years)	57.2 ± 10.5
Sex (*n*, men)	6
Ethnicity (*n*, non-Hispanic/Hispanic)	8/2
Race (*n*, Caucasian/Other)	8/2
Body mass index (kg·m^−2^)	30.6 ± 4.3
Waist circumference (cm)	99.8 ± 7.2
VO_2_peak (mL·kg^−1^·min^−1^)	28.5 ± 5.8
Systolic blood pressure (mmHg)	136.5 ± 12.1
Diastolic blood pressure (mmHg)	83.4 ± 6.7
Heart rate (bpm)	71.4 ± 8.2
Duration of hypertension (years)	6.3 ± 5.7
Medication use (%) ^1^	40.0

Note: VO_2_peak, maximal oxygen consumption. ^1^ Medication was discontinued for the study duration.

**Table 2:** The descriptive characteristics of the aerobic exercise training program (mean ± SD).

Frequency (session/week)	2.8 ± 0.5
Intensity ^1^	
Heart rate (bpm)	128.5 ± 13.1
Heart rate reserve (%)	62.4 ± 9.0
Rating of perceived exertion	13 ± 0.1
Training impulse	52.9 ± 18.4
Heart rate max (%)	78.5 ± 4.2
Time (min/session) ^1,2^	35.9 ± 6.9
Type	
Treadmill modality (%)	93.7 ± 18.1

^1^ Average over 12 weeks of exercise training. ^2^ Excluding 5 min warmup and 5 min cool down.

## Data Availability

Supporting data can be found within the article and Supplementary Materials. Additional data that are not reported are available upon request from the corresponding author.

## Supplementary Materials

The following supporting information can be downloaded at https://www.mdpi.com/article/10.3390/jcdd11020042/s1, Formulas S1: Repeated measures models, AIC, and ICC [41,42]; Figure S1: Plot of residuals under RMANCOVA; Figure S2: Plot of residuals under GRMANCOVA; Table S1: Results of repeated measures models for the continuous blood pressure and binary postexercise hypotension outcomes.
