# Supplementary material for: Reliability and Time Course of Postexercise Hypotension during Exercise Training among Adults with Hypertension
Source: J Cardiovasc Dev Dis. 2024 Jan 29;11(2):42. doi: 10.3390/jcdd11020042 (PMC10889392; doi:10.3390/jcdd11020042)
Supplement: Supplementary file 1 [file jcdd-11-00042-s001.zip › Kiernan Supplemental Codes S1 12-27-23.pdf]

Supplemental Codes S1: R-codes.

R codes for RMANCOVA and GRMANCOVA model are provided. Codes for the other two models are similar.

```
# RMANCOVA
library(lme4)
library(car)
library(dplyr)
mod_c <- lmer(y ~ week + SBPBASE + DBPBASE + (1|ID), REML=F, data=df)
## calculate AIC
AIC(mod_c)
## Type III ANOVA table
Anova(mod_c, type = "III")
smry_c <- summary(mod_c)
## between subject
sig_alpha_sq <- smry_c$varcor$ID[1]
## random error
sig_sq <- smry_c$sigma^2
ICC_c <- sig_alpha_sq / (sig_alpha_sq + sig_sq)

# GRMANCOVA
mod_b <- glmer(y ~ week + SBPBASE +DBPBASE+ (1|ID), family = binomial(link = "logit"),
        data=df,control=glmerControl(optimizer="bobyqa",optCtrl=list(maxfun=2e4)))
smry_b <-summary(mod_b)
AIC(mod_b)
## coefficients
coef_b <- smry_b$coefficients[,1]
## variance estimate
var_b <- smry_b$varcor$ID[1]
## design matrix
xmat <- model.matrix(~1+week + SBPBASE + DBPBASE, df)
## linear predictor
xb <- data.frame(xb=xmat %*% coef_b)
post <- df %>% bind_cols(xb2)
```

```
# integrand of variance for week j
sigmaj <- function(x) {
  pj=0
  for (i in 1:n) {
    pj=pj+ 1/(1+exp(-xb[i]-x)) / n
  }
  f=pj*(1-pj)*(1/sqrt(2*pi*sig) )*exp(-0.5*x^2 /sig)
  return(f)
}
# integrand of first part in \sigma_{\tau}^2
sumpj1 <- function(x) {
  pj=0
  for (j in 1:12) {
    data_sub <- post %>% filter(week==j)
    # n: number of observations in jth week
    n <- data_sub$n1[1]
    xb <- data_sub$xb
    sig <- var_b
    for (i in 1:n) {
      pj=pj+ 1/(1+exp(-xb[i]-x)) / n
    }
  }
  f=pj^2 * (1/sqrt(2*pi*sig))*exp(-0.5*x^2 /sig)
  return(f)
}
# integrand of second part in \sigma_{\tau}^2
sumpj2 <- function(x) {
  pj=0
  for (j in 1:12) {
    data_sub <- post %>% filter(week==j)
    n <- data_sub$n1[1]
    xb <- data_sub$xb
    sig <- var_b
    for (i in 1:n) {
```

```
      pj=pj+ 1/(1+exp(-xb[i]-x)) / n
    }
  }
  f=pj * (1/sqrt(2*pi*sig))*exp(-0.5*x^2 /sig)
  return(f)
}
a <- integrate(sumpj1, -Inf, Inf)$value
b <- integrate(sumpj2, -Inf, Inf)$value
sig_tau_sq <- a-b^2

# J needs to be assigned with a value
sig <- vector()
for (j in 1:J) {
  data_sub <- post %>% filter(week==j)
  n <- data_sub$n1[1]
  xb <- data_sub$xb
  sig <- var_b
  sig[j] = integrate(sigmaj, -Inf, Inf)$value
}
sig_e_sq <- sum(sig)
icc_b <- sig_tau_sq/(sig_tau_sq+sig_e_sq)
```
