# Supplementary material for: Reliability and Time Course of Postexercise Hypotension during Exercise Training among Adults with Hypertension
Source: J Cardiovasc Dev Dis. 2024 Jan 29;11(2):42. doi: 10.3390/jcdd11020042 (PMC10889392; doi:10.3390/jcdd11020042)
Supplement: Supplementary file 1 [file jcdd-11-00042-s001.zip › Kiernan Supplemental Formulas 1-24-24.pdf]

Supplemental Formulas S1: Repeated Measures Models, AIC, and ICC

Repeated Measures Models

Assume that there are N subjects involved in the J weeks project, and within each week each individual attends K sessions. Let $\mathrm{y_{ijk}}$, $\mathrm{b_i}$ and $\mathrm{week_j}$ denote the continuous BP response, subject effect and week effect respectively from the ith subject in the jth week and kth session for $i = 1, \cdots, N$, $j = 1, \cdots, J$ and $k = 1, \cdots, K$. In addition, let $\mathrm{SBPBASE_{ijk}}$ and $\mathrm{DBPBASE_{ijk}}$ denote the SBP and DBP values at the beginning of the jth week and kth session for the ith subject with β_1 and β_2 being the corresponding coefficients. Four different models are applied under different settings

RMANOVA

$$\mathrm{y_{ijk}} = \mathrm{b_i} + \mathrm{week_j} + \mathrm{e_{ijk}},$$

$$\mathrm{b_i} \sim \mathrm{N}(0, \sigma_b^2),$$

$$\mathrm{e_{ijk}} \sim \mathrm{N}(0, \sigma_e^2),$$

where y_{ijk} is a continuous variable which takes the lower value of SBP relative change and DBP relative change and e_{ijk} is the experimental error for $i = 1, \cdots, N$, $j = 1, \cdots, J$ and $k = 1, \cdots, K$.

RMANCOVA

$$\mathrm{y_{ijk}} = \beta_1 \times \mathrm{SBPBASE_{ijk}} + \beta_2 \times \mathrm{DBPBASE_{ijk}} + \mathrm{b_i} + \mathrm{week_j} + \mathrm{e_{ijk}},$$

$$\mathrm{b_i} \sim \mathrm{N}(0, \sigma_b^2),$$

$$\mathrm{e_{ijk}} \sim \mathrm{N}(0, \sigma_e^2),$$

where all terms are defined in RMANOVA.

GRMANOVA

$$\mathrm{logit}(\mathrm{p_{ijk}}) = \log\left(\mathrm{p_{ijk}}/(1 - \mathrm{p_{ijk}})\right) = \mathrm{b_i} + \mathrm{week_j},$$

$$\mathrm{y_{ijk}} \sim \mathrm{Bernoulli}(\mathrm{p_{ijk}}),$$

$$\mathrm{b_i} \sim \mathrm{N}(0, \sigma^2\ ),$$

where y_{ijk} is the binary PEH response which takes value 1 when subject i experiences a decrease in either SBP or DBP in the jth week and kth session, and 0 otherwise for $i = 1, \cdots, N$, $j = 1, \cdots, J$ and $k = 1, \cdots, K$.

GRMANCOVA

$$\mathrm{logit}(\mathrm{p_{ijk}}) = \beta_1 \times \mathrm{SBPBASE_{ijk}} + \beta_2 \times \mathrm{DBPBASE_{ijk}} + \mathrm{b_i} + \mathrm{week_j},$$

$$\mathrm{y_{ijk}} \sim \mathrm{Bernoulli}(\mathrm{p_{ijk}}),$$

$$\mathrm{b_i} \sim \mathrm{N}(0, \sigma^2\ ),$$

where y_{ijk} and β_0 are defined in GRMANOVA.

AIC

AIC assessed the goodness-of-fit of models and serves as one of the major tools when performing model selection. The formula of AIC is given by Akaike (2),

AIC=-2 log-maximum likelihood + 2p

where p is the number of model parameters. Model with smaller AIC is preferred.

ICC

For the continuous BP response, ICC is a commonly used measurement to quantify the strength of how two observations are related within the same group, which can be expressed as

$$\mathrm{ICC} = \frac{\sigma_b^2}{\sigma_b^2 + \sigma_e^2},$$

where all terms are defined in the Repeated Measures Models part.

In terms of binary PEH response, there are various methods proposed to calculate ICC. Inspired by Dimitrov (1), we developed a new method to compute ICC for a binary response.

For a single observation, given α_i, we have

$$\mu_j(\alpha_i) = \tau_j(\alpha_i) + e_j(\alpha_i),$$

where $\mu_j(\alpha)$ is the binary score and $\tau_j(\alpha_i)$ represents the true score for the jth week, and $e_j(\alpha_i)$ denotes the random error. Thus, we have

$$\sigma^2(e_j(\alpha_i)) = \sigma^2(\mu_j(\alpha_i)) = p_j(\alpha_i)(1 - p_j(\alpha_i))$$

Let $\mathbf{x_{ijk}} = (SBPBASE_{ijk}, DBPBASE_{ijk})'$ and $\beta = (\beta_1, \beta_2)'$. Furthermore, $\mathbf{x_j} = \{\mathbf{x_{ilk}}\}_{l=j}$. Assume that

$$\mathbf{x_j} \sim f(\mathbf{x}),$$

then $p_j(\alpha_i)$ can be estimated with the following marginal probability density function:

$$p_j(\alpha_i) = \int_{\mathbf{x}} \frac{exp(\mathbf{x_j}'\boldsymbol{\beta} + \alpha_i + \mathrm{week_j})}{1 + exp(\mathbf{x_j}'\boldsymbol{\beta} + \alpha_i + \mathrm{week_j})} f(\mathbf{x})\mathrm{d}\mathbf{x}.$$

Let n_j denote the number of observations, and $(n_{j1}, \cdots, n_{jl_j})$ denote frequency of $(w_{j1}, \cdots, w_{jl_j})$, which is the set of distinct values of $\mathbf{x_{ijk}}$ for the jth week, where l_j is the length of the set such that $\sum_{m=1}^{l_j} n_{jm} = n_j$.

Therefore, the empirical evaluation of $p_j(\alpha_i)$ is

$$p_j^*(\alpha_i) = \sum_{m=1}^{l_j} \frac{n_{jm}}{n_j} \frac{exp(\mathbf{w_{jm}}'\boldsymbol{\beta} + \alpha_i + \mathrm{week}_j)}{1 + exp(\mathbf{w_{jm}}'\boldsymbol{\beta} + \alpha_i + \mathrm{week}_j)}.$$

Variance for week j has the expression form as

$$\sigma^2(e_j) = \int_{-\infty}^{\infty} p_j^*(\alpha)(1 - p_j^*(\alpha)) g(\alpha)\mathrm{d}\alpha,$$

where $g(\alpha)$ is the probability density function of α_i.

Hence, assuming independence of the random errors, we have

$$\sigma_e^2 = \sum_{j=1}^{J} \sigma^2(e_j).$$

Let $\dot{p} = \sum_{j=1}^{J} p_j^*$, σ_τ^2 has the following expression (3),

$$\sigma_{\mathrm{b}}^2 = \int_{-\infty}^{\infty} \dot{p}^2\, g(\alpha)\mathrm{d}\alpha - [\int_{-\infty}^{\infty} \dot{p}\, g(\alpha)\mathrm{d}\alpha]^2.$$

Finally, the ICC for a binary response model can be estimated by

$$\mathrm{ICC} = \frac{\sigma_{\mathrm{b}}^2}{\sigma_{\mathrm{b}}^2 + \sigma_{\mathrm{e}}^2}.$$

References

1. Dimitrov DM. Marginal true-score measures and reliability for binary items as a function of their IRT parameters. Applied Psychological Measurement. 2003;27(6):440-58.

2. Akaike H. A new look at the statistical model identification. IEEE Transactions on Automatic Control. 1974;19(6):716-23.

3. May K, Nicewander WA. Reliability and Information Functions for Percentile Ranks. Journal of Educational Measurement. 1994;31(4):313-25.
