# Supplementary material for: Reliability and Time Course of Postexercise Hypotension during Exercise Training among Adults with Hypertension
Source: J Cardiovasc Dev Dis. 2024 Jan 29;11(2):42. doi: 10.3390/jcdd11020042 (PMC10889392; doi:10.3390/jcdd11020042)
Supplement: Supplementary file 1 [file jcdd-11-00042-s001.zip › Kiernan Supplemental Table 12-27-23.pdf]

Table S1. Results of Repeated Measures Models for the Continuous Blood Pressure and Binary Postexercise Hypotension Outcomes

Model	Variable	Chisq	Df	P-value	AIC	ICC
RMANOVA	Week	4.1685	11	0.9647	2248.5202	0.3076
	Week	13.7314	11	0.2482		
RMANCOVA	Pre-exercise SBP	49.6335	1	0.0000	2177.9031	0.5834
	Pre-exercise DBP	3.6928	1	0.0547		
GRMANOVA	Week	4.4145	11	0.9562	230.2550	0.3422
GRMANCOVA	Week	5.1847	11	0.9219	224.9167	0.6361

***Note*:** AIC = akaike information criterion, Chisq = chi square, Df = degrees of freedom, GRMANOVA = generalized repeated measures analysis of variance, GRMANCOVA = generalized repeated measures analysis of covariance, ICC = intraclass correlation coefficient, RMANOVA=repeated measures analysis of variance, RMANCOVA = repeated measures analysis of covariance
