# Supplementary figures and images for: Reliability and Time Course of Postexercise Hypotension during Exercise Training among Adults with Hypertension
Source: J Cardiovasc Dev Dis. 2024 Jan 29;11(2):42. doi: 10.3390/jcdd11020042 (PMC10889392; doi:10.3390/jcdd11020042)

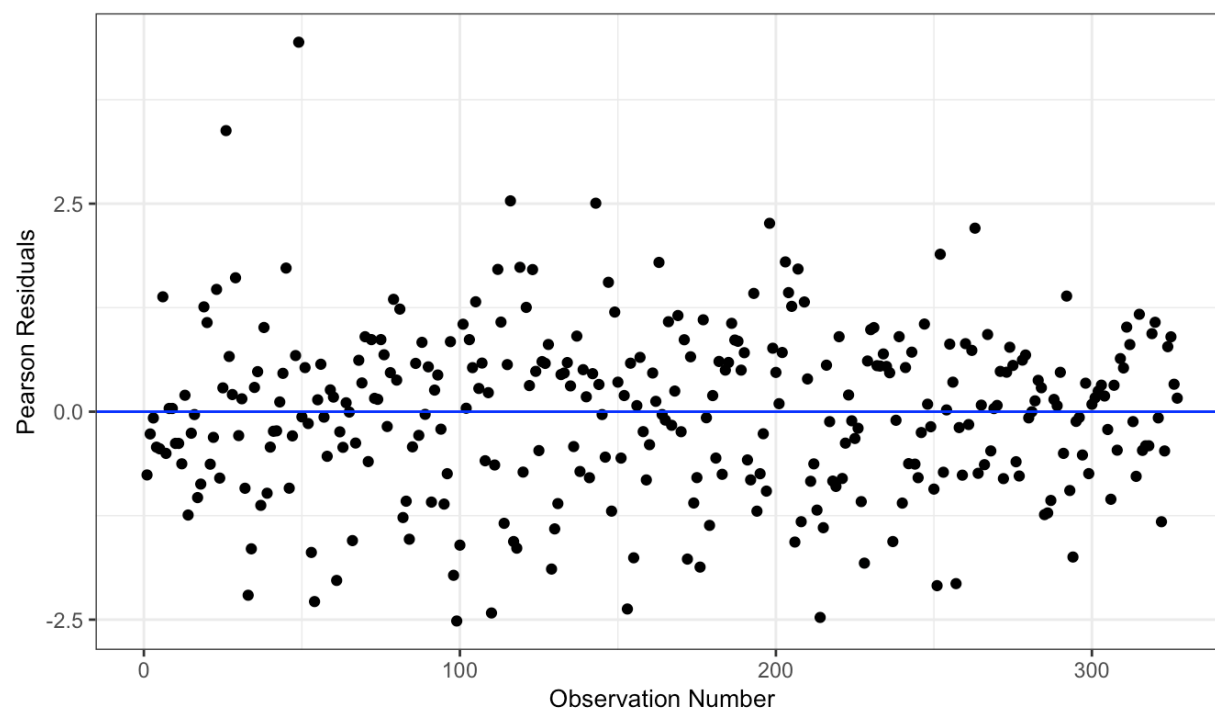

**Figure S1.** Plot of Residuals Under RMANCOVA

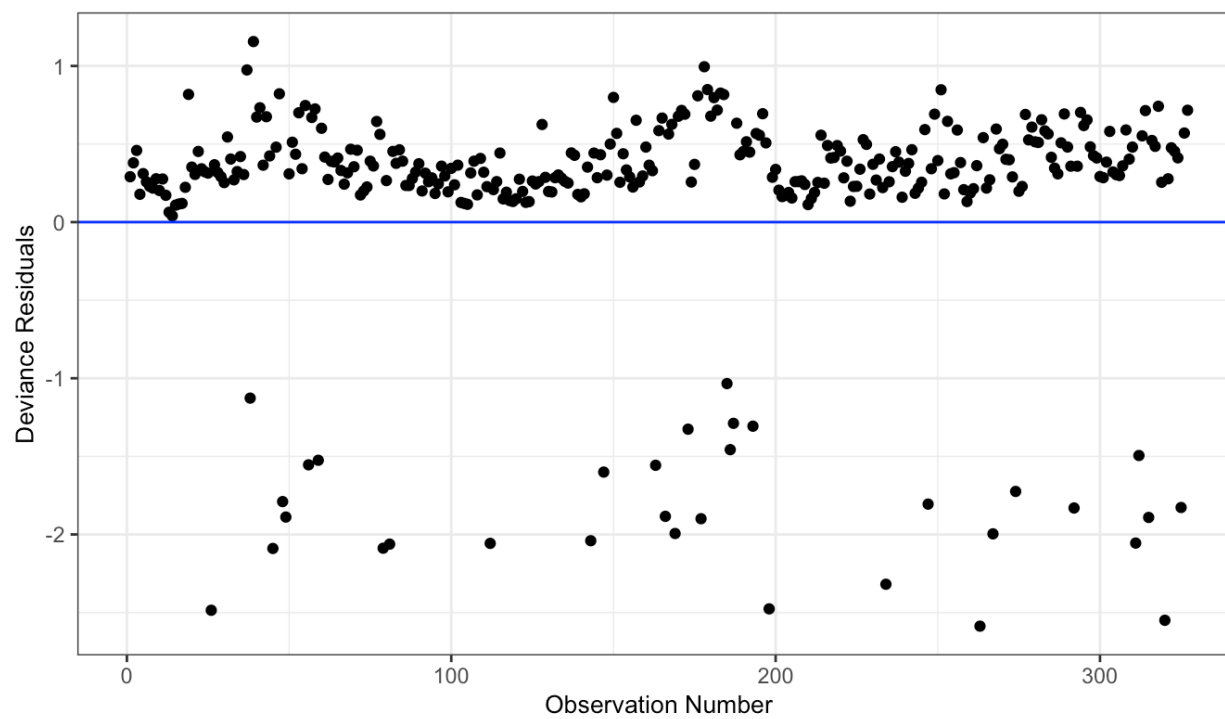

**Figure S2.** Plot of Residuals Under GRMANCOVA

Supplement: Supplementary file 1 [file jcdd-11-00042-s001.zip › Kiernan Supplemental Graphs 12-27-23.pdf]
